# Influence of border disease virus (BDV) on serological surveillance within the bovine virus diarrhea (BVD) eradication program in Switzerland

**DOI:** 10.1186/s12917-016-0932-0

**Published:** 2017-01-13

**Authors:** V. Kaiser, L. Nebel, G. Schüpbach-Regula, R. G. Zanoni, M. Schweizer

**Affiliations:** 1 Institute of Virology and Immunology, Federal Food Safety and Veterinary Office (FSVO) and Vetsuisse Faculty, University of Bern, Laenggass-Strasse 122, POB, CH-3001 Bern, Switzerland; 2 Veterinary Public Health Institute, Vetsuisse Faculty, University of Bern, Schwarzenburgstrasse 155, CH-3097 Liebefeld, Switzerland

**Keywords:** Bovine viral diarrhea virus (BVDV), Border disease virus (BDV), Pestivirus, Serum neutralisation test (SNT), Seroprevalence, Small ruminants, Cross-neutralisation, Eradication, Risk factor

## Abstract

**Background:**

In 2008, a program to eradicate bovine virus diarrhea (BVD) in cattle in Switzerland was initiated. After targeted elimination of persistently infected animals that represent the main virus reservoir, the absence of BVD is surveilled serologically since 2012. In view of steadily decreasing pestivirus seroprevalence in the cattle population, the susceptibility for (re-) infection by border disease (BD) virus mainly from small ruminants increases. Due to serological cross-reactivity of pestiviruses, serological surveillance of BVD by ELISA does not distinguish between BVD and BD virus as source of infection.

**Results:**

In this work the cross-serum neutralisation test (SNT) procedure was adapted to the epidemiological situation in Switzerland by the use of three pestiviruses, i.e., strains representing the subgenotype BVDV-1a, BVDV-1h and BDSwiss-a, for adequate differentiation between BVDV and BDV. Thereby the BDV-seroprevalence in seropositive cattle in Switzerland was determined for the first time. Out of 1,555 seropositive blood samples taken from cattle in the frame of the surveillance program, a total of 104 samples (6.7%) reacted with significantly higher titers against BDV than BVDV. These samples originated from 65 farms and encompassed 15 different cantons with the highest BDV-seroprevalence found in Central Switzerland. On the base of epidemiological information collected by questionnaire in case- and control farms, common housing of cattle and sheep was identified as the most significant risk factor for BDV infection in cattle by logistic regression.

**Conclusion:**

This indicates that pestiviruses from sheep should be considered as a source of infection of domestic cattle and might well impede serological BVD surveillance.

**Electronic supplementary material:**

The online version of this article (doi:10.1186/s12917-016-0932-0) contains supplementary material, which is available to authorized users.

## Background

The genus *Pestivirus* in the family *Flaviviridae* comprises the four established species border disease virus (BDV), bovine viral diarrhea virus type-1 (BVDV-1), bovine viral diarrhea virus type-2 (BVDV-2) and classical swine fever virus (CSFV). Additional putative pestivirus species were isolated from giraffe (“Giraffe-1 pestivirus”), cattle (“atypical pestiviruses”), antelopes (“Pronghorn antelope pestivirus ”) und piglets (“Bungowannah virus”) [1–3]. Recently, an additional new strain termed “atypical porcine pestivirus” was isolated from pigs and piglets with congenital tremor [4, 5]. The ruminant pestiviruses BVDV and BDV are important pathogens with a worldwide distribution [6] causing substantial economic losses in farm animal husbandry [7, 8].

Acute, transient infections of seronegative, immunocompetent animals with ruminant pestiviruses are frequently asymptomatic or are accompanied by mild respiratory or enteric symptoms [9, 10]. By contrast, acute infection of pregnant cattle between approx. day 40 to 120 of gestation may cause transplacental transmission of non-cytopathogenic (ncp) biotypes to the fetus leading to the birth of persistently infected (PI) calves. These animals shed virus life-long and, thereby, comprise the primary pestivirus reservoir [11–13]. Similarly, lambs persistently infected with border disease virus caused by transplacental transmission display alterations in their fleece and show tremor (hence there are also called ‘hairy shakers’), and they may succumb by a syndrome resembling Mucosal disease in cattle [14–17].

BDV in small ruminants occurs worldwide but with very variable seroprevalence depending, e.g., on the geographic location and the type of animal husbandry [14, 17–20]. It was for the first time isolated in Switzerland in a flock of sheep that gave birth to lambs with generalized tremors and excessively hairy fleece in 2001 [21]. In a study published in 1995, seroprevalence in registered sheep flocks of breeding associations and in large flocks was around 20 and 65%, respectively [22]. More recent data pointed to a slightly lower seroprevalence of 13.5% [23] or 16.1% [18] in sheep and 25.4% in goats [18]. In the latter study, it was demonstrated by means of cross-serum neutralisation tests (cross-SNT) that 9% of the sheep and 6% of the goats were infected with BDV. However, 31% and 66% of the seropositive sheep and goats, respectively, could not be assigned to BVDV or BDV leaving the source of infection unidentified.

Thus, even though broad serological cross-reactivity occurs among pestiviruses, considerable quantitative differences in neutralisation efficiency can be measured by SNT between different species (also called genotype) [24–26] and even subgenotypes [26–33]. To date, up to 21 (1a to 1u) and three (2a to 2c) subgenotypes were described within the pestivirus species BVDV-1 and BVDV-2, respectively [34, 35], mostly based on comparison of the 5′-UTR (untranslated region) or N^pro^ (N-terminal protease) region of the pestiviral genomes. In Switzerland, BVDV-1e, -1h, -1k, and -1b are the most prevalent subgenotypes identified in cattle, while BVDV-2 was never detected [30, 36, 37]. Similarly, BD viruses exhibit a large heterogeneity of strains [38] with 7 main subgenotypes and several atypical BDV strains described [39]. An additional phylogenetic group was detected in our institute exclusively in Switzerland and provisionally named BD Switzerland or BDSwiss [40, 41]. Lately, a similar isolate was identified in Italy and labeled as BDV-8 [42].

As ruminant pestiviruses are not strictly species specific, they are able to infect a variety of even-toed ungulates (Artiodactyla) [3, 13, 20]. Virus transmission was described between cattle and both, sheep and goats ([19, 43, 44], and references therein). Natural infections of cattle with BDV were reported in England and Wales [45, 46], Austria [47, 48], Italy [49] and New Zealand [50]. Common housing of cattle with persistently infected sheep was the most important cause for seroconversions, and resulted in reduced fertility and abortions in pregnant cows [19, 44, 46]. BDV-specific seroconversion in cattle was reported in Switzerland after pasturing them with BDV-positive sheep on common alpine meadows [51].

Based on the economic impact of BVDV infections in livestock, several European countries therefore initiated programs to eradicate BVDV in cattle [52, 53]. In Switzerland, such a program was started in 2008 that particularly targeted on a nationwide identification and elimination of PI animals [54, 55]. Initially, 1.4% of all newborn calves were persistently infected with BVDV, which dropped to less than 0.02% by the end of 2012. From that time on, BVD control is based predominantly on risk-based serological surveillance of bulk milk and blood samples [56], with continuously decreasing seroprevalence. As serology performed by ELISA does not distinguish between an infection with BVDV from one with BDV, the impact of infection with BDV on serological surveillance on BVDV is not known. Thus, the aim of this study was to determine the frequency of BDV infections in cattle by using an optimized cross-neutralisation SNT, to identify potential risk factors for interspecies transmission with special emphasis on small ruminants, and to assess their possible influence on the serological surveillance of BVD in bovines.

## Methods

### Sera

Sera used in this study were from the period 2012 to 2014 and were initially rated as “indeterminate” or “positive” to antibodies (Ab) against pestivirus by ELISA performed by a primary laboratory and later confirmed as positive by the national BVD reference laboratory (Institute of Veterinary Virology/Institute of Virology and Immunology, Bern, Switzerland). A serum was confirmed as positive when it was either positive in the institutes “in house”-ELISA [57] or when it was rated positive in a serum neutralisation test (SNT) using BVDV-1a (Table 1) as challenge virus. Sera were only included if they were obtained from animals that were at least 6 months old at the time of sampling and that were born later than Sept. 30, 2009, i.e., after phase 2 of the Swiss BVD eradication program [58]. If several samples from the same animal were obtained, only the one that was analysed first in the reference laboratory was used for the analysis. All sera were stored at -20 °C prior to use. Overall, 1,568 sera fulfilled the criteria mentioned above, with 506, 536, and 526 sera obtained in the years 2012, 2013, and 2014, respectively.

**Table 1:** Ruminant pestivirus isolates selected for SNT

Pestivirus	Subgenotype	Isolate	Species	Source^d^	Reference
BDV	Swiss-a	R9336/11	Cattle	IVV/IVI BE	[40]
BDV	Swiss-b	R4785/06/CH-BD4	Sheep	IVV/IVI BE	[44]
BDV	3	R1343/01/CH-BD1	Sheep	IVV/IVI BE	[36]
BDV	1a	Moredun	Sheep	P. Nettleton^a^	[72]
BVDV-1	1h	CH-04-01b	Cattle	IVV/IVI BE	[30]
BVDV-1	1e	CH-Maria	Cattle	IVV/IVI BE	[30]
BVDV-1	1k	CH-Suwa (ncp)^c^	Cattle	IVV/IVI BE	[30]
BVDV-1	1b	CH-04-05	Cattle	IVV/IVI BE	[30]
BVDV-1	1a	R1935/72 (cp)^c^	Cattle	IVV/IVI BE	[59]
BVDV-2	2a	890	Cattle	J. F. Ridpath^b^	[73]

^a^Moredun Research Institute, Edinburgh, Scotland

^b^National Animal Disease Center, Ames IA, USA

^c^ *cp* cytopathogenic, *ncp* non-cytopathogenic

^d^IVV/IVI BE = Institute of Veterinary Virology/Institute of Virology and Immunology, Bern

### Cells

Bovine turbinate (BT) cells were prepared at the Institute of Veterinary Virology (University of Bern, Switzerland) from bovine fetuses obtained from a local abattoir and were maintained in Earle’s minimal essential medium (E-MEM; Biochrom GmbH, Berlin, Germany) supplemented with 15% fetal calf serum (FCS) (2% during experiments), 100 U/ml penicillin, and 100 μg/ml streptomycin at 37 °C in a humidified 5% CO_2_ atmosphere. FCS was free of pestivirus and antibodies to BVDV/BDV as tested by virus isolation and SNT, respectively. BT cells were found to be free of pestivirus by immunoperoxidase staining, and they were used to produce virus stocks, and to perform SNTs and virus (back-)titrations.

### Production of challenge viruses used for SNT

Overall, 10 strains of ruminant pestiviruses from different subgenotypes were selected as challenge virus for the SNT (4× BDV, 5× BVDV-1 and 1× BVDV-2) (Table 1). All major subgenotypes hitherto isolated in Switzerland were represented by one isolate (BDSwiss-a, BDSwiss-b, BDV-3 and BVDV-1h, -1e, -1k, -1b) [37, 40]. With the exception of the North American strain Oregon C24 (R1935/72, BVDV-1a, [59]), all isolates were of the non-cytopathogenic (ncp) biotype. Each isolate was propagated in 150 cm^2^ cell culture flasks (TPP AG, Trasadingen, Switzerland) seeded with 3 × 10^6^ BT cells in 50 ml E-MEM with 15% FCS. One day post-seeding, the cells were infected for 1 h at a multiplicity of infection (moi) of 0.01 in 10 ml E-MEM with 7% FCS followed by the addition of 40 ml of E-MEM with 7% FCS. Cell infected with an ncp biotype of pestivirus were further incubated for 5 days at 37 °C and 5% CO_2_, whereas the cells infected with the cytopathogenic (cp) BVDV-1a strain were harvested at the time when 80% of the cells showed signs of cytopathic effect (CPE) as judged by light microscopy. Virus stocks were obtained by freeze-thawing the cells at -20 °C and removal of cell debris by centrifugation for 15 min at 10,000g (HiCen® 21C, Hemotec GmbH, Gelterkinden, Switzerland). Aliquots of 0.5 ml were stored at -80 °C until use.

### Homologous sera

For each of the 10 subgenotypes, a serum as homologous as possible was chosen. Eight out of 10 sera were Swiss field sera, whereas the immune sera to the genotypes BVDV-1a (strain R1935/72) and BVDV-2 were obtained from vaccine trials in Switzerland and Germany, respectively. The latter was raised against the BVDV-2 strain CS8644 [60] and was kindly provided by G. Wolf (LMU, Munich, Germany). For the subgenotype BDV-1a that was never detected in Switzerland to date, no homologous serum was available and, thus, we used a field serum from an antibody-positive heifer with unknown source of infection that displayed a rather high titer against BDV-1a. All sera were stored at -20 °C.

### Serum neutralisation test

For the detection and quantification of pestivirus-specific neutralising antibodies in cattle sera, a serum neutralisation test (SNT), which is considered the gold standard in BVDV serology [61, 62], was developed and optimized for the current situation in Switzerland. Basically, the SNT was done according to the directions of the OIE [63]. Briefly, sera to be tested were pre-diluted tenfold in E-MEM with 2% FCS and inactivated for 30 min at 56 °C. In the cases were only an insufficient amount of serum was available for all experiments, the serum was pre-diluted 20- (*n* = 46) or 40-fold (*n* = 6). Thereafter, sera were further diluted seven times in twofold steps up to a dilution of 1 in 1,280. Dilutions were directly done in 96-well plates with 4 wells per dilution and in a volume of 50 μl of serum per well. Afterwards, 100 tissue culture infectious dose 50 (TCID_50_) of challenge virus in 50 μl E-MEM with 2% FCS per well were added and the plates were incubated for one hour at 37 °C in a humidified 5% CO_2_ atmosphere. Subsequently, a cell suspension in E-MEM with 2% FCS with 20,000 cells in a volume of 100 μl was added to each well and further incubated for 4 to 5 days. For each experiment, a positive (α-BVDV-1b) and a negative control serum was included, and to control for possible serum cytotoxicity, 50 μl of serum of the first pre-dilution was added to another well in the absence of challenge virus. For evaluation of the ncp strains, immunoperoxidase staining was performed, whereas the neutralisation of the cp strain was directly quantified by analysis of the CPE by light microscopy. The neutralisation titer was calculated according to Spearman-Kaerber and expressed as reciprocal value of the dilution required for 50% of the wells exhibiting neutralisation of the challenge virus. Samples with a titer greater than 8 were rated as positive.

In order to differentiate the source of infection, we performed cross-neutralisation tests using different strains of ruminant pestiviruses as challenge virus in parallel SNTs. To identify the strains with the best discriminatory power, we screened various combinations of BVDV and BDV as challenge virus, whereby an at least fourfold difference of their SNT titers were regarded as significant [63]. Taking the samples with low volume (20-fold pre-dilution) into account, sera with BVDV and BDV titers lower than 15 were regarded as negative. A ratio of the BVDV- and BDV-titer of lower than 4 was considered as “indeterminate”.

### Virus titration

Virus titrations were performed according to the direction of the OIE [63]. Briefly, the virus suspensions were diluted seven times in tenfold steps in E-MEM with 2% FCS directly in 96-well plates with 6 wells per dilution using a volume of 50 μl per well. Six wells were used as cell control with the simple addition of medium. After addition of the cell suspension (20,000 cells per well in 100 μl E-MEM with 2% FCS), the plates were incubated for 4 to 5 days at 37 °C in a humidified 5% CO_2_ atmosphere. Virus titers were calculated according to Spearman-Kaerber and presented as tissue culture infectious dose 50 (TCID_50_) per ml. In every SNT, the amount of challenge virus applied (100 TCID_50_) was controlled by back-titration in parallel to the SNT, and a variation of half a log level was considered as acceptable (10^1.5^–10^2.5^ TCID_50_ = 32 to 316) [63]. SNTs with the back-titration being outside of this range were repeated.

### Immunoperoxidase staining

An immunoperoxidase staining was applied for cells infected with an ncp biotype of pestivirus. Cells were washed with PBS and subsequently thoroughly dried for at least one hour in the air flow of a safety cabinet. Thereafter, cells were fixed and permeabilised by incubation for two hours at 80 °C. After the plates regained room temperature, the primary antibody (polyclonal swine-α-BVDV hyperimmune serum prepared at the Institute of Veterinary Virology, University of Bern) was applied at a dilution of 1 to 750 in PBS with 5% Tween-20 (PBS-T) and incubated for 90 min at ambient temperature. After washing the cells three times with PBS-T, the secondary antibody (monoclonal peroxidase-labeled goat-α-swine IgG; KPL, Gaithersburg, MD, USA) at a dilution of 1 to 1,000 in PBS-T with 5% low-fat powdered milk was applied for 90 min at room temperature. Subsequently, cells were washed twice with PBS-T and once with distilled water, followed by the addition of the substrate solution (0.4 mg/ml 3-Amino-9-ethylcarbazole (AEC), 6% dimethylformamide, 0.3‰ H_2_O_2_ in 0.05 M sodium acetate at pH 5.0) and incubation for approx. 30 min until an adequate staining was observed. Staining was stopped by washing the cells with distilled water.

### Case-control-study

In order to detect risk factors for infection with border disease virus in livestock, we performed a retrospective case-control-study in farms whose young stock was surveyed serologically in the BVD eradication program in Switzerland and the Principality of Liechtenstein [64]. Young stocks are used for sero-surveillance in non-dairy herds. Each year, a random sample of one third of all farms that did not have a PI animal within the last 24 months, and all herds that either had a PI animal or a conspicuous result in bulk milk testing within this time period, are tested serologically. A group of young stock is tested that consists of 5 calves within a herd of an age of at least 6 months, which were born later than September 30, 2009 and more than one month after the removal of the last known PI animal from the farm. In addition, these calves should have stayed on a given farm for at least 6 months and not previously had a known contact with a PI animal. Based on these criteria, 54 farms with at least two seropositive animals in the time interval from February 2014 to June 2015 were selected as potential case farms. Sera from such potential case farms were tested in the cross-SNT, and the farm was considered a definite case farm if the herd contained at least one single seropositive animal that was rated as infected with BDV. Potential case farms which proved negative for BDV in the cross-SNT were excluded. Control farms were selected out of a pool of 10,753 farms (data collection by the Swiss Veterinary Service) that were never tested seropositive for BVDV since the start of the eradication program in 2008 and that had tested at least 5 animals as seronegative since the beginning of 2012. Control farms were selected using the Swiss animal movement database [56]. Because only 16 definitive case farms that also returned the questionnaire (see below) could be identified, the number of controls was increased to reach a relation of at least three controls per case.

### Data acquisition

To identify potential risk factors for the occurrence of BDV infections in cattle, variables such as presence of small ruminants, herd size, use and distribution of breed, animal movement (summer pasturing), common housing and pasture management, and external contact to (wild) ruminants, were collected by a standardized, bipartite questionnaire for cattle farmers. The first part of the questionnaire contained a total of 55 questions regarding the farm itself, contacts to animals on neighboring farms, or purchase and sale (animal movement) with special emphasis on possible direct and indirect exposure to small ruminants. The second part mainly aimed at receiving information on the origin of seropositive and seronegative animals in case and control farms, respectively. Additionally, information on the breeds of the animals was obtained from the animal movement database. The questionnaires were issued in German, French and Italian, and were distributed to the Cantonal Veterinary Services starting in the middle of August 2014. Official veterinarians or the farm veterinarians were instructed by the Cantonal Veterinary Services to perform the interviews that were conducted on-site or by phone.

### Statistics

Data of the SNTs and the questionnaires were entered into the spreadsheet Excel® 2010 (Microsoft Corporation). The frequency distribution of the different potential risk factors in the 37 case and 280 control animals from 16 case and 56 control farms were statistically compared using the Chi-Square test for categorical variables and with the two-sample *t*-Test for normally distributed continuous variables, whereas the Mann-Whitney-*U*-Test was used for not normally distributed variables. After the univariable analysis of each individual risk factor according to the questionnaire, a multivariable logistic regression model with the potential risk factors (*p* < 0.1) was developed. In the model, clustering on the level of the farm was corrected with the GEE (generalized estimating equation) method [65]. None of the 13 identified potential risk factors were highly correlated with each other (correlation coefficient <0.7), therefore all of them were offered to the model. The final model was established by stepwise forward selection of the risk factors, until only significant factors (*p* < 0.05) and confounders remained in the model. Risk factors were considered as confounders and kept in the model if they altered the regression coefficient of another risk factor by more than 25%. The univariable and multivariable statistical analysis of the data was performed with NCSS (version 9; NCSS LLC, Kaysville UT) and with PROC GENMOD in SAS (version 9.4; SAS Institute Inc., Cary NC), respectively. Model fit was assessed with QIC and QICu fit criteria, as well as with visual assessment of the residuals.

## Results

### Distribution of field sera

**Fig. 1:**
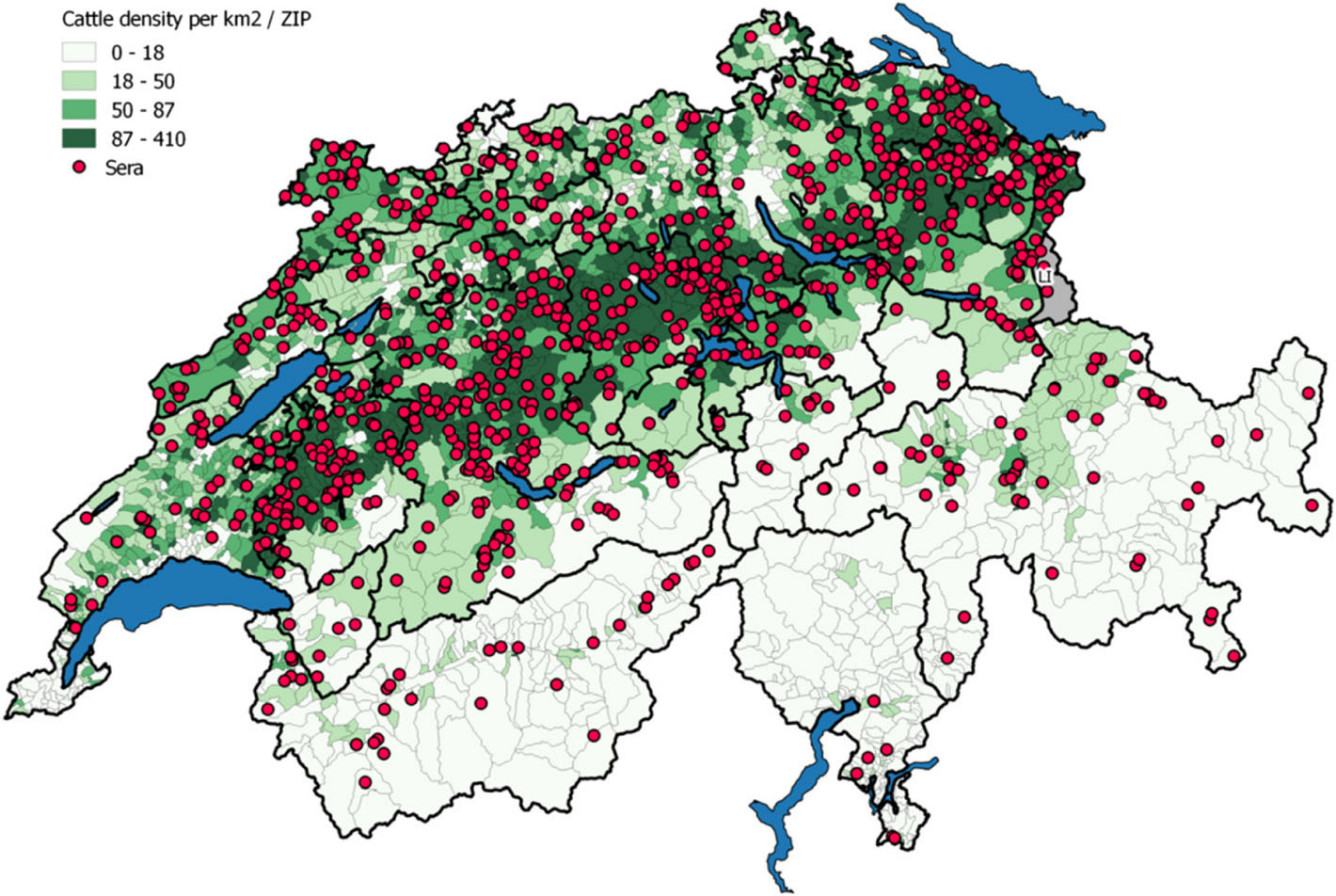
Geographic distribution of field sera and density of the cattle population in Switzerland and of the Principality of Liechtenstein (LI). The map was created with the geographic information system QGIS 2.8.1-Wien. The number of cattle per ZIP (postal code; data 2014) were obtained from the Swiss Federal Statistical Office (https://www.pxweb.bfs.admin.ch); n (Farms providing sera) = 898

Around three quarters (74.8%) of the 1,568 confirmed antibody-positive sera (see Methods section) were from cattle that were born prior to 2012 with 44% (*n* = 512) thereof in the year 2010. Animals at the age of 6 to 11 months at the time of examination represented the largest age group (*n* = 347, 22.1%) which is in accordance with the specifications of the «spot test» [55]. Overall, samples from 24 out of the 26 Swiss (half-) cantons and of the Principality of Liechtenstein were at our disposition, and the sample distribution was in good agreement (Fig. 1) with the cattle density per km^2^ per locality (based on postal code) in Switzerland.

### Cross-neutralisation of the selected BDV and BVD strains

For the selection of the most appropriate BD and BVD virus strains to be used in the cross-SNT, we tested all possible combinations of the 10 isolates of ruminant pestiviruses (BDV, BVDV-1, BVDV-2; Table 1). For each subgenotype, we selected a serum as homologous as possible and titrated them in the SNT with all the pestivirus strains (see Additional file 1: Table S1). With only few exceptions, the highest titers were detected with the homologues pairs of virus and serum. The highest titer of 5,120 was observed with the anti-BVDV-1e serum and the corresponding virus strain CH-Maria. The only exceptions were the sera raised against BDSwiss-a, BDV-1a, and BVDV-1k, which reacted equally or even stronger in response to other virus isolates than their homologous virus strain. As expected [26, 66], the anti-BVDV-2a serum exhibited a low (average titer = 48) and a medium (average titer = 135) neutralisation reactivity against BDV and BVDV-1, respectively. Conversely, the anti-BDV sera poorly (mean titer = 19) and moderately (mean titer = 164) neutralized the BVDV-2a strain 890 and the BVDV-1 strains, respectively. All BVDV-1 sera except the one directed against BVDV-1h only weakly neutralized BD viruses (mean titer = 47), whereas their cross-neutralisation was rather high against heterologous BVDV-1 strains (mean titer = 523). Finally, sera directed against BDV showed a low and medium neutralisation activity towards BVDV-1 strains (mean titer = 57) and heterologous BD viruses (mean titer = 192), respectively.

### Coefficients of antigenic similarity (R)

The antigenic relatedness of a pair of viruses was determined by calculating the coefficient of antigenic similarity (R) [67] according to the following formula:

$$ \mathrm{R} = 100 \times \sqrt{\frac{\ \mathrm{titer}\ \mathrm{virus}\ \mathrm{isolate}\ \mathrm{A}\ \mathrm{with}\ \mathrm{serum}\ \mathrm{B} \times \mathrm{titer}\ \mathrm{virus}\ \mathrm{isolate}\ \mathrm{B}\ \mathrm{with}\ \mathrm{serum}\ \mathrm{A}}{\mathrm{titer}\ \mathrm{virus}\ \mathrm{isolate}\ \mathrm{A}\ \mathrm{with}\ \mathrm{serum}\ \mathrm{A} \times \mathrm{titer}\ \mathrm{virus}\ \mathrm{isolate}\ \mathrm{B}\ \mathrm{with}\ \mathrm{serum}\ \mathrm{B}}} $$

A value of R ≤ 25 is considered to denote a significant antigenic difference between the virus strains [24, 30]. All possible combinations of BD and BVD viruses displayed an *R*-value lower than 25, which is indicative of a significant antigenic difference between two strains. The largest differences with *R*-values lower than two were observed between BDV and BVDV-1 strains, whereas the four strains of BDV displayed the highest *R*-values among each other (Table 2) with no significant difference between any two strains.

**Table 2:** Coefficients of antigenic similarity *(R)* between BDV and BVDV isolates

BDV & BVDV isolates	BDV Swiss-a	BDV Swiss-b	BDV−3	BDV-1a	BVDV−1h	BVDV−1e	BVDV−1k	BVDV−1b	BVDV−1a	BVDV−2a
BDSwiss-a	**100**	77.1	40.3	71.0	*19.2*	*≤ 2.3*	*≤ 13.6*	*6.0*	*≤ 2.2*	*4.8*
BDSwiss-b		**100**	29.8	73.8	*10.1*	*1.8*	*19.2*	*5.5*	*1.6*	*3.0*
BDV-3			**100**	59.5	*14.8*	*3.7*	*15.6*	*5.5*	*3.0*	*3.4*
BDV-1a				**100**	*20.1*	*4.8*	*≤ 23.8*	*12.0*	*2.5*	*6.7*
BVDV-1h					**100**	*13.6*	52.3	32.4	*12.5*	*11.5*
BVDV-1e						**100**	*22.9*	*13.1*	*7.1*	*6.5*
BVDV-1k							**100**	52.3	28.5	*11.5*
BVDV-1b								**100**	*21.0*	*5.0*
BVDV-1a									**100**	*3.6*
BVDV-2a										**100**

*R*-Values ≤ 25 indicate significant antigenic differences between two isolates (in italics). For nondescript titers (≤ 14), the value of 14 was taken for calculation and, accordingly, the *R*-values are marked with “≤”

### Selection of the pestivirus strains used in cross-neutralisation of field sera

The quotients of titers of the cross-neutralisation assays of the 10 sera with the four BD and the six BVD viruses were calculated for all possible pairwise combinations (Table 3), with the absolute numbers of the SN titers listed in supplementary Additional file 1: Table S1. Values of the quotient below 4 were considered as indeterminate. All antisera against BDV were correctly assigned with 4 different combinations of virus pairs (BVDV-1a and BDSwiss-a; BVDV-2a and BDSwiss-a or BDSwiss-b; BVDV-1k and BDSwiss-a). The six BVDV antisera were correctly classified in three pairwise combinations of the subgenotype BVDV-1h, e.g., with the strains BDSwiss-a, BDSwiss-b and BDV-3. Overall, the virus pair BVDV-1a and BDSwiss-a displayed the best differentiation of all sera, with correct classification of 8 out of 10 sera in the cross-neutralisation assays. By adding the results of the BVDV-1h strain CH-04-01b in combination with the BDV strain Swiss-a, both remaining BVD-antisera (anti-BVDV-1h and anti-BVDV-2) could be properly assigned. Therefore, the pestivirus strains BVDV-1a, BVDV-1h and BDSwiss-a were selected as being optimally suited for the differentiation between BVDV and BDV as source of infection in the current epidemiological situation in Switzerland.

**Table 3:** Ratios of the cross-SNT titers of 10 sera tested with all combinations of BDV and BVDV isolates

Sera	Virus pair											
	BVDV-1h				BVDV-1e				BVDV-1k			
	BD Swiss-a	BD Swiss-b	BDV-3	BDV-1a	BD Swiss-a	BD Swiss-b	BDV-3	BDV-1a	BD Swiss-a	BD Swiss-b	BDV-3	BDV-1a
α-BDSwiss-a	4.4	4.4	**1.9**	**2.0**	5.1	5.1	**2.2**	**2.4**	8.8	8.8	**3.7**	4.1
α-BDSwiss-b	**4.0** ^a^	6.7	**1.8**	**2.2**	5.6	9.5	**2.6**	**3.1**	8.8	14.7	4.0	4.8
α-BDV-3	**1.1**	**0.9**	**2.8**	**0.9**	**1.5**	**1.3**	4.0	**1.3**	4.4	**3.7**	11.4	**3.7**
α-BDV-1a	**1.7**	**2.6**	**1.7**	**1.5**	**3.1**	4.8	**3.1**	**2.8**	11.4	17.6	11.4	10.5
α-BVDV-1h	6.2	14.7	16.0	16.0	**0.6**	**1.5**	**1.7**	**1.7**	**0.5**	**1.1**	**1.2**	**1.2**
α-BVDV-1e	64.6	60.3	32.3	26.6	365.7	341.3	182.9	150.6	11.4	10.7	5.7	4.7
α-BVDV-1k	22.7	6.8	13.3	6.2	10.4	**3.1**	6.1	**2.8**	6.2	**1.8**	**3.6**	**1.7**
α-BVDV-1b	22.7	19.0	26.7	16.0	10.4	8.7	12.2	7.3	4.8	4.0	5.6	**3.4**
α-BVDV-1a	38.1	10.4	15.9	24.5	17.5	4.8	7.3	11.3	19.1	5.2	7.9	12.3
α-BVDV-2a	8.0	4.3	8.0	**3.4**	7.3	**4.0** ^a^	7.3	**3.1**	**1.7**	**0.9**	**1.7**	**0.7**
Sera	Virus pair											
	BVDV-1b				BVDV-1a				BVDV-2a			
	BD Swiss-a	BD Swiss-b	BDV-3	BDV-1a	BD Swiss-a	BD Swiss-b	BDV-3	BDV-1a	BD Swiss-a	BD Swiss-b	BDV-3	BDV-1a
α-BDSwiss-a	5.1	5.1	**2.2**	**2.4**	8.8	8.8	**3.7**	4.1	8.2	8.2	**3.5**	**3.8**
α-BDSwiss-b	4.3	7.3	**2.0**	**2.4**	34.5	58.1	15.8	18.8	22.6	38.0	10.3	12.3
α-BDV-3	**2.0**	**1.7**	5.2	**1.7**	4.4	**3.7**	11.4	**3.7**	6.2	5.2	16.0	5.2
α-BDV-1a	**2.0**	**3.1**	**2.0**	**1.8**	11.4	17.6	11.4	10.5	10.7	16.5	10.7	9.8
α-BVDV-1h	**1.5**	**3.7**	4.0	4.0	**0.6**	**1.4**	**1.5**	**1.5**	**0.5**	**1.3**	**1.4**	**1.4**
α-BVDV-1e	32.4	30.2	16.2	13.3	24.9	23.3	12.5	10.3	11.4	10.7	5.7	4.7
α-BVDV-1k	19.1	5.7	11.2	5.2	6.2	**1.8**	**3.6**	**1.7**	**2.6**	**0.8**	**1.5**	**0.7**
α-BVDV-1b	54.0	45.3	63.5	38.1	8.0	6.7	9.4	5.7	**3.7**	**3.1**	4.3	**2.6**
α-BVDV-1a	69.8	19.1	29.1	45.0	234.8	64.3	97.8	151.5	5.7	**1.5**	**2.4**	**3.6**
α-BVDV-2a	**2.0**	**1.1**	**2.0**	**0.8**	**2.8**	**1.5**	**2.8**	**1.2**	53.5	29.1	53.5	22.7

For the calculation of the ratios of the α-BD sera, the titer of BDV was taken as numerator and the titer of BVDV as denominator. For the calculation of the ratios of the α-BVD sera the reverse ratio was used. Ratios < 4 are highlighted in bold

^a^The exact value was slightly below 4.0

### Neutralisation and cross-neutralisation of field sera

In order to differentiate BVDV and BDV as source of infection, we employed the cross-SNT with the three challenge strains BVDV-1a, BVDV-1h, and BDSwiss-a as described above. From 1,568 samples that were initially confirmed to be positive for pestivirus antibodies and that originated from 898 farms, we were able to analyze 1,555 in the cross-SNT. This includes the six results to the BVDV-1a strain from the initial SNT that was applied by the reference laboratory for samples where not sufficient material was available to perform full cross-neutralisation.

**Fig. 2:**
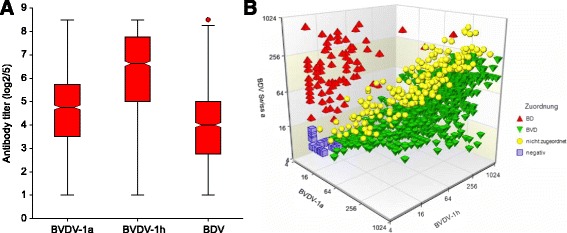
Distribution of SNT titers of sera against BVDV-1a, BVDV-1h and BDV. **a** The box plots of titers of positive sera were created with the statistical software NCSS 9. Only positive titers (> 8) were included (n_BVDV-1a_ = 1,400; n_BVDV-1h_ = 1,496; n_BDV_ = 1,292). For titers with a value of ≤ 14 (20-fold pre-dilution), ≤ 28 (40-fold pre-dilution) and ≥ 1,810, the values 14, 28 and 1,810 were taken for calculation, respectively. The SNT titers are represented in the y-axis as logarithm to the base 2 including the standard 5-fold pre-dilution, i.e., multiplication with 5 yields the final titer. **b** The scatter plot of the sera samples (*n* = 1,555) based on their titers against BDSwiss-a, BVDV-1a and BVDV-1h was created with the statistical software NCSS 9. The black dotted line represents the threefold rotational axis on which each of the three titers per sample would have the same value and, therefore, no assignment to “BVD” or “BD” is possible (*yellow*). BDV-specific sera with low reaction against BVDV-1a are located in the upper left section of the cube (*red*), whereas BVDV-specific sera with high reaction against BVDV-1h are located in the lower right section of the cube (*green*). Negative sera are in the lower left corner (*purple*)

Both, the average titer (geometric mean titer (GMT) = 346.3; median = 495.5) of the field sera and the neutralisation titer of the majority of the individual samples (1,394 out of 1,549 samples) were higher towards the BVDV-1h isolate than the ones towards the BVDV-1a strain (GMT = 125.5; median = 135). The average (GMT = 74.8; median = 80) as well as most of the single titers to BDSwiss-a were clearly lower than the values to the BVD viruses (Fig. 2a). The highest titer (≥1,810) was reached with 153, 14, and one sera against BVDV-1h, BVDV-1a, and BDV, respectively. Most sera with negative neutralisation titers (≤ 8) were observed using BDV in the SNT (*n* = 257).

### Identification of the source of infection by cross-SNT

By combining the results of the SNTs with the virus strains of the subgenotype BVDV-1a and BDSwiss-a, we were able to differentiate the source of infection between BVDV and BDV in 550 out of 1,555 samples (35.4%). By adding the BVD/BD-quotient with the BVDV-1h strain to the remaining samples (i.e., 870 samples that could not be assigned using only the BVDV-1a strain, and to 135 samples that were rated as negative), further 666 sera could be sourced to an infection with BVDV (Table 4). In 93% of all BVD-positive cases, the sera had a higher titer towards BVDV-1h than BVDV-1a. Conversely, for samples that were assigned to an infection with BDV, their low reactivity towards the BVDV-1a strain was pivotal for their differentiation from BVDV (Fig. 2b). In no instance a contradictory combination of the results was observed. Overall, the majority of samples were assigned to BVD (*n* = 1,112, 71.5%; CI 95%: 69.2–73.7%), and only 104 cattle sera (6.7%; CI 95%: 5.5–8.0%) were attributed to an infection with BDV. In 28 samples that could initially not be differentiated because they still exhibit full neutralisation even at the highest dilution, repetition of the experiments with more dilutions steps allowed their differentiation to an infection with BVDV (*n* = 27) or BDV (*n* = 1). The remaining sera could either not be differentiated (*n* = 286, 18.4%; CI 95%: 16.5–20.4%) or were regarded as negative (*n* = 53, 3.4%; CI 95% 2.6–4.4%) based on their low titers (titers ≤ 14).

**Table 4:** Combinations of results from the cross-SNT tested with 3 isolates

Evaluation with BVDV-1a & BDV		Evaluation with BVDV-1h & BDV	Assignment^a^	*n* =	Percentage of combination per assignment [%]	Proportion overall [%]
BVD	&	BVD	BVD	428	38.5	71.5
BVD	&	Negative		4	0.4	
BVD	&	Indeterminate		13	1.2	
Indeterminate	&	BVD		594	53.5	
Negative	&	BVD		72	6.5	
BVD		n.d.^b^		1	0.1	
BD	&	BD	BD	68	65.4	6.7
BD	&	Indeterminate		32	30.8	
BD		n.d.^b^		1	3.8	
Indeterminate	&	Indeterminate	Indeterminate	276	96.5	18.4
Negative	&	Indeterminate		10	3.5	
Negative	&	Negative	Negative	52	98.1	3.4
Negative		n.d.^b^		1	1.9	
Total				1,555		100

^a^Sera with a ratio of ≥ 4 were assigned as “BVD” or “BD”, sera with a ratio < 4 were “indeterminate”, and sera with both titers < 15 are “negative”

^b^ *n.d* not done, due to insufficient amount of material

### BDV as source of infection: distribution of samples

**Fig. 3:**
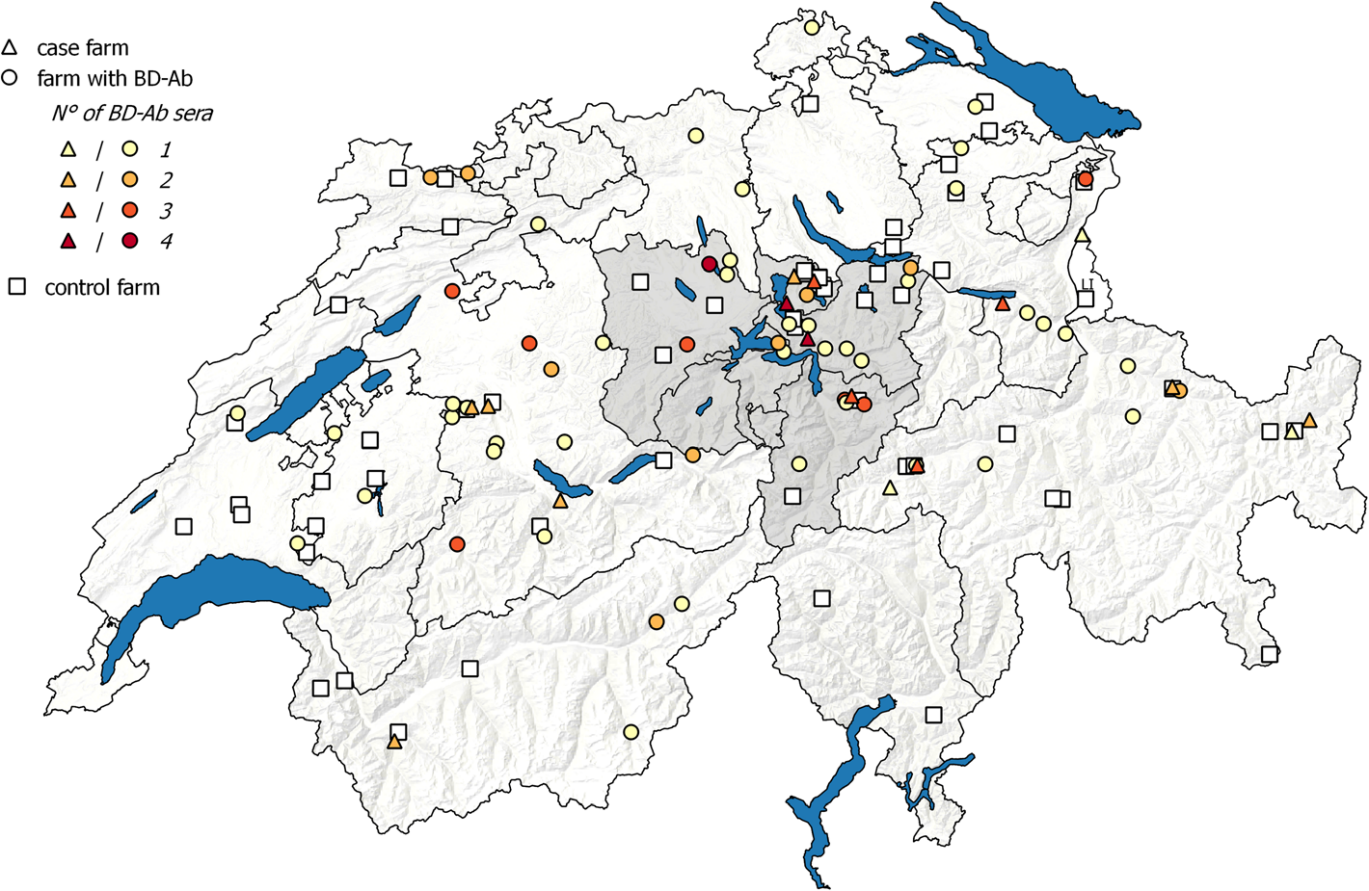
Farms with BDV-positive sera and case and control farms in Switzerland and the Principality of Liechtenstein (LI). The location of farms with BDV-positive sera (*circles*) including the definitive case farms (*triangle*) and the control farms (*squares*) are presented. The number of BDV-specific sera from one to four is displayed by a color gradient from yellow to red as indicated in the figure

The 104 cattle sera that were assigned to BDV as source of infection originated from 65 farms within 15 cantons (Fig. 3). A large part of the samples came from Central Switzerland (*n* = 36), in accordance with the observation that the two cantons with the highest prevalence of BDV-reactive sera (64.7 and 32.4%) were from this area. Notably, in three of these farms in Central Switzerland, calves that were persistently infected with BDV were detected within the scope of the Swiss BVD eradication program (H.P. Stalder, personal communication and [37]). In 9 cantons and in the Principality of Liechtenstein, we could not detect any BDV positive sample within the 206 sera that we analyzed.

In 21 out of 29 farms where a BDV-specific sample and more than one positive serum in the cross-SNT was obtained, all of the samples were specific for BDV as source of infection. From the remaining farms, four had samples that could not be differentiated or were negative, whereas in the other four, BVDV could also be identified as source, sometimes in combination with indeterminate samples.

According to information retrieved from the Swiss animal movement database [58], small ruminants were present on 44 farms (68%) where BDV was identified as source of infection in at least one sample. Thereof, 27 (61%) of these farms housed sheep, 4 (9%) goats, and 13 (30%) both, sheep and goats. By contrast, only a third of the farms without BDV-positive samples (*n* = 259, 31%) kept small ruminants according to information obtained from the animal movement database.

### Selection of farms for the case-control-study

We obtained detailed data on 54 potential case farms that were recruited between February 2014 and June 2015 based on the serological screening results using a standardized questionnaire. BDV was later identified by means of cross-SNT as the source of infection in 37 samples from 16 farms that were distributed across 7 cantons and the Principality of Liechtenstein (Fig. 3). Additional 3 samples from these definitive case farms were indeterminate and one was negative. In no case farm could we detect any BVDV-specific serum.

### Descriptive, univariable statistics

By means of Pearsons’s chi square test, we identified significant differences (*p* < 0.1) in 9 out of 50 categorical variables and, using Mann-Whitney-*U*-test or *t*-test, in 4 out of 5 continuous variables (Additional file 2: Table S2a and S2b). The variables sheep farming, sheep breed, and origin of cattle display the lowest *p* values (*p* < 0.0001). Within the continuous variables, the number of cattle was considerably (*p* = 0.0006) and the number of sheep, goats, and loss of lambs was fairly different (*p* = 0.1) between the case and control groups (Additional file 3: Table S3).

### Logistic regression

Variables that were significant according to the univariable analysis and additional variables of epidemiological relevance regarding animal movement (purchase, provenance) were introduced into the model of logistic regression by stepwise forward selection. At the farm level, the risk factors “same stable” (sheep and cattle are kept in the same stable; OR (odds ratio) = 167.23, CI_95%_: 15.37–1,819.29, *p* < 0.0001) and “cattle purchase” (purchase of cattle within the last 12 months; OR = 9.57, CI_95%_: 1.08–84.95; *p* = 0.0426), and at the level of the individual animal, the risk factor “cattle provenance” (cattle was purchased; OR = 4.16, CI_95%_: 1.64–10.60; *p* = 0.0028) could be confirmed as significant in the final model.

## Discussion

In this study, we investigated the frequency of BDV infections in cattle and evaluated their possible influence on the serological surveillance in the Swiss BVD eradication program. Thus, we selected one strain out of each major subgenotype of ruminant pestiviruses detected in Switzerland so far [37] in addition to the two strains previously used for differentiation in routine diagnostic, i.e., R1935/72 (BVDV-1a) and Moredun (BDV-1a). In order to choose the most appropriate challenge viruses in the cross-SNT to differentiate BVDV from BDV infections, all possible pairwise combinations out of the 6 BVDV and 4 BDV strains were tested (Table 1) together with sera that were as homologous as possible to the corresponding virus isolates. The combination of BVDV-1a and BDSwiss-a turned out to be an optimal combination that correctly assigned 8 out of 10 test sera (Table 3), including all BDV-specific samples, and that yielded the highest ratios of the neutralisation titers (mean = 33.6). In addition, by analyzing the effect of varying the dose of challenge virus employed in the SNT (10^2^ TCID_50_ ± 0.5 log as acceptable range for the challenge viruses) on the SN titers essentially confirmed the usefulness of a quotient of four used in classic serology as a threshold for significance (not shown). And notably, the use of cytopathogenic strains such as the strain R1935 (BVDV-1a) was advantageous as it allowed for direct microscopical evaluation of the results. In field situations, however, only non-cytopathogenic strains are available and, thus, the need for fixation and immunostaining of the cells cannot be avoided.

In contrast to the test sera, the combination of BVDV-1a and BDSwiss-a was not able to differentiate between BVDV and BDV as the source of infection in more than half of the field sera (55.9%) (Table 4). In addition to the BVDV-1a subgenotype, which was never found to circulate in Switzerland, we thus included a strain of the in our country most commonly found genotype BVDV-1h [30, 37] into the analysis. Using these three strains as challenge viruses in the cross-SNT, we were able to diminish the rate of indeterminate samples to 18.4%. As a result, we could considerably improve the former triage which was used in the laboratory that applied only two strains (BVDV-1a and BDV-1a Moredun) and that was thus unable to assign 31.0 and 66.4% of the sheep and goat sera, respectively [18]. In the case of a BVDV infection, we generally observed higher neutralisation titers against the challenge virus BVDV-1h than towards BVDV-1a, which is based on the higher antigenic homology of the field sera to a strain circulating in the Swiss cattle population. But the exclusive use of the BVDV-1h strain in combination with BDSwiss-a was not favorable as it only poorly identified BDV infections. Based on the quotients of the neutralisation titers observed (Table 3), further reduction of the number of indeterminate sera proved to be difficult. In case of acute infections in the field, direct virus isolation and partial sequencing of the viral genome would be required [37]. Nonetheless, the adaptation of the serological triage system to the current epidemiological situation proved to be an important prerequisite for optimal selectivity to differentiate between ruminant pestivirus infections.

A small number of samples (3.4%) that were initially confirmed to be antibody positive by ELISA could not be confirmed in the cross-SNT (Table 4). In spite of the significant correlation between the SNT and the ELISA results (not shown), the two assays are inherently different. Thus, the epitopes important for the neutralisation assay are mainly part of the E2 envelope glycoprotein in its native conformation, whereas the “in-house” ELISA detects primarily antibodies directed to the conserved non-structural protein NS2-3 [57, 62, 68].

The results of the cross-SNTs comprising the three challenge viruses BVDV-1a, BVDV-1h, and BDSwiss-a, clearly demonstrated that the majority of pestivirus infections in cattle discovered 4 to 7 years after the start of the eradication can be ascribed to BVDV (71.5%), whereas only 6.7% were caused by infections with BD viruses. Nevertheless, it has to be considered that at least some of the indeterminate sera (18.4%) might also have BDV as source of infection. The BDV seroprevalence within the pestivirus-antibody positive animals slightly increased between 2012 and 2014 from 4.2 to 8.1% (not shown), whereas the overall seroprevalence in the Swiss cattle population continuously decreases [69]. These results point to a potential interference by small ruminants, in particular sheep, with the eradication of BVDV from cattle. Interestingly, small ruminants were kept – according to the entries in the animal movement database – on all case farms and on the majority of farms where BDV infections were detected by serology, with sheep being more prominent than goats. Notably, sheep flocks with more than 100 animals were exclusively found within the case farms. The highest portion of BDV-specific sera (*n* = 36; 34.6%) as well as the highest BDV seroprevalence (19% of all seropositive samples) were obtained from Central Switzerland (Fig. 3) while the number of animals and of samples in this region are within average of all greater areas of Switzerland (Fig. 1).

By means of a logistic regression model to determine the influence of potential risk factors for an infection with BDV, we provided evidence for the impact of small ruminants. Common indoor housing of sheep and cattle was identified as the risk factor with the highest odds ratio in the final model (OR = 167.23). Further variables with significant but smaller influence were the purchase of cattle on the level of the farm (purchase within the last 12 month) (OR = 9.57) and on the level of the individual animal (OR = 4.16). As the sample size of seropositive farms was limited, the power of the study was not sufficient to identify risk factors with weak association. Nonetheless, the final model confirms previous reports that pointed to the relevance of common housing of sheep and cattle as a main risk factor [19, 23, 44, 70]. In particular, sheep persistently infected with BDV pose a considerable risk when housed together with BVDV-free livestock. In the course of a BVD eradication scheme, the importance of interspecies transmission might increase with the continuous decrease in antibody seroprevalence in cattle and their ensuing increase in susceptibility to (re-)infection with pestiviruses [19].

## Conclusion

Collectively, our study proposes that farmers with common housing of cattle and sheep should be aware of interspecies virus transmission, especially during lambing, where a high infection pressure exists [71]. In situations where contact between cattle and sheep cannot be avoided or minimized, surveillance of pestiviruses in sheep might be considered [46]. The Swiss eradication program encompasses only bovines, but not sheep and goats. Thus, the mean BDV seroprevalence in pestivirus-antibody positive cattle of at least 6.7% with an increasing trend between 2012 and 2014 indicates that the serological surveillance by ELISA, which does not differentiate BVDV from BDV infections, might be critical. Even though discrimination by cross-SNT as described in this study is laborious, it adds to classical epidemiological investigations and allows the identification of possible sources of infection, which is of particular importance in the late phase of an eradication program [37, 48]. In summary, we determined for the first time the prevalence of BDV in pestivirus-positive cattle in Switzerland, and we provide strong evidence that common housing of cattle and sheep is the most significant risk factor for the interspecies transmission of BD virus from small ruminants to cattle.

## Additional files

Additional file 1: Antibody titer of 10 sera against homologous and heterologous BDV and BVDV isolates. Cross-neutralisation titers of 10 sera against five, four, and one BVDV-I, BDV, and BVDV-II strain, respectively. (DOCX 19 kb)
Additional file 2: Categorical risk factors with significant differences. Categorical risk factors with significant differences between case (BDV-seropositive) and control (seronegative) farms (Additional file 2: Table S2a) and, on the animal level, between case (BDV-seropositive) and control (seronegative) farms (Additional file 2 Table S2b). (DOCX 23 kb)
Additional file 3: Continuous risk factors with significant differences between case (BDV-seropositive) and control (seronegative) farms. Within the continuous variables, differences between the number of cattle, sheep, goats, and loss of lambs between the case and control groups. (DOCX 21 kb)

## Abbreviations

Ab: Antibody
AEC: 3-amino-9-ethylcarbazole
BDV: Border disease virus
BT cells: Bovine turbinate cells
BVDV: Bovine virus diarrhea virus
CI: Confidence interval
cp: Cytopathogenic
CSFV: Classical swine fever virus
ELISA: Enzyme-linked immunosorbent assay
E-MEM: Earle’s minimal essential medium
FCS: Fetal calf serum
moi: Multiplicity of infection
n.d: Not done
ncp: Non-cytopathogenic
N^pro^: N-terminal protease
OIE: Office International des Epizooties (World Organisation for Animal Health)
OR: Odds ratio
PBS: Phosphate buffered saline
PBS-T: PBS with 5% Tween-20
PI: Persistently infected
SNT: Serum neutralisation test
TCID_50_: Tissue culture infectious dose 50
UTR: Untranslated region
