# Supplementary material for: Influence of border disease virus (BDV) on serological surveillance within the bovine virus diarrhea (BVD) eradication program in Switzerland
Source: BMC Vet Res. 2017 Jan 13;13:21. doi: 10.1186/s12917-016-0932-0 (PMC5237232; doi:10.1186/s12917-016-0932-0)
Supplement: Additional file 1: — Antibody titer of 10 sera against homologous and heterologous BDV and BVDV isolates. Cross-neutralisation titers of 10 sera against five, four, and one BVDV-I, BDV, and BVDV-II strain, respectively. (DOCX 19 kb) [file 12917_2016_932_MOESM1_ESM.docx]

Table S1: Antibody titer of 10 sera against homologous and heterologous BDV and BVDV isolates

| ↓Sera / Isolates🡪 | BDSwiss-a | BDSwiss-b | BDV-3 | BDV-1a | BVDV-1h | BVDV-1e | BVDV-1k | BVDV-1b | BVDV-1a | BVDV-2a |
| --- | --- | --- | --- | --- | --- | --- | --- | --- | --- | --- |
| α-BDV-Swiss-a | **123** | 123 | 52 | 57 | 28 | 24 | ≤ 14 | 24 | ≤ 14 | 15 |
| α-BDV-Swiss-b | 587 | **987** | 269 | 320 | 147 | 104 | 67 | 135 | 17 | 26 |
| α-BDV-3 | 123 | 104 | **320** | 104 | 113 | 80 | 28 | 62 | 28 | 20 |
| α-BDV-1a | 160 | 247 | 160 | **147** | 95 | 52 | ≤ 14 | 80 | ≤ 14 | 15 |
| α-BVDV-1h | 453 | 190 | 174 | 174 | **2,792** | 293 | 207 | 698 | 269 | 247 |
| α-BVDV-1e | ≤ 14 | 15 | 28 | 34 | 905 | **5,120** | 160 | 453 | 349 | 160 |
| α-BVDV-1k | 20 | 67 | 34 | 73 | 453 | 207 | **123** | 381 | 123 | 52 |
| α-BVDV-1b | 67 | 80 | 57 | 95 | 1,522 | 698 | 320 | **3,620** | 538 | 247 |
| α-BVDV-1a | 20 | 73 | 48 | 31 | 761 | 349 | 381 | 1,396 | **4,695** | 113 |
| α-BVDV-2a | 31 | 57 | 31 | 73 | 247 | 226 | 52 | 62 | 87 | **1,660** |

Homologous titers are in bold.
