# Supplementary material for: Influence of border disease virus (BDV) on serological surveillance within the bovine virus diarrhea (BVD) eradication program in Switzerland
Source: BMC Vet Res. 2017 Jan 13;13:21. doi: 10.1186/s12917-016-0932-0 (PMC5237232; doi:10.1186/s12917-016-0932-0)
Supplement: Additional file 2: — Categorical risk factors with significant differences. Categorical risk factors with significant differences between case (BDV-seropositive) and control (seronegative) farms (Additional file 2: Table S2a) and, on the animal level, between case (BDV-seropositive) and control (seronegative) farms (Additional file 2 Table S2b). (DOCX 23 kb) [file 12917_2016_932_MOESM2_ESM.docx]

Table S2a: Categorical risk factors with significant differences between case (BDV-seropositive) and control (seronegative) farms

| Risk factors | Description | Category | Case (n = 16) | Control (n = 56) | P-value^1)^ |
| --- | --- | --- | --- | --- | --- |
| Sheep farming | Keeping of sheep on the farm | Yes | 14 | 9 | < 0.0001 |
|  |  | No | 2 | 47 |  |
| Sheep breed | Breed of the sheep | WAS^2)^ | 10 | 6 | < 0.0001 |
|  |  | Other | 4 | 3 |  |
|  |  | No sheep | 2 | 47 |  |
| Same stable | Sheep and cattle are kept in the same stable | Yes | 11 | 3 | 0.0300 |
|  |  | No | 3 | 6 |  |
| Housing systems | Housing systems of sheep in the same stable with cattle | Common stabling | 0 | 1 | 0.0469 |
|  |  | Separate boxes | 11 | 2 |  |
| Contact-rating | Assessment of the contact between sheep and cattle by the owner | Frequent / intense | 6 | 0 | 0.0312 |
|  |  | Rare / low | 5 | 8 |  |
|  |  | n/a ^2)^ | 3 | 1 |  |
| Goats | Keeping of goats | Yes | 8 | 9 | 0.0048 |
|  |  | No | 8 | 47 |  |
| Neighboring pasture | Contact on pasture with foreign small ruminants | Yes | 2 | 3 | 0.0912 |
|  |  | No | 12 | 52 |  |
|  |  | n/a ^2)^ | 2 | 1 |  |

*^1)^ p-value < 0.1, calculated with Pearson’s Chi-Square test*

^2)^ *WAS = White Alpine Sheep; n/a = not applicable*

Table S2b: Categorical risk factors on animal level with significant differences between case (BDV-seropositive) and control (seronegative) farms

| Risk factors | Description | Category | Case (n = 37) | Control (n = 280) | P-value ^1)^ |
| --- | --- | --- | --- | --- | --- |
| Cattle breed | Breed of the cattle by type of use | Dairy | 19 | 138 | 0.0665 |
|  |  | Meat & other breed | 15 | 77 |  |
|  |  | Dairy & Meat | 3 | 65 |  |
| Cattle origin | Birth on farm or purchased | Farm | 19 | 228 | < 0.0001 |
|  |  | Purchase | 18 | 52 |  |

^1)^ p-value < 0.1, calculated with Pearson’s Chi-Square test
