# Supplementary material for: Influence of border disease virus (BDV) on serological surveillance within the bovine virus diarrhea (BVD) eradication program in Switzerland
Source: BMC Vet Res. 2017 Jan 13;13:21. doi: 10.1186/s12917-016-0932-0 (PMC5237232; doi:10.1186/s12917-016-0932-0)
Supplement: Additional file 3: — Continuous risk factors with significant differences between case (BDV-seropositive) and control (seronegative) farms. Within the continuous variables, differences between the number of cattle, sheep, goats, and loss of lambs between the case and control groups. (DOCX 21 kb) [file 12917_2016_932_MOESM3_ESM.docx]

Table S3: Continuous risk factors with significant differences between case (BDV-seropositive) and control (seronegative) farms

| Risk factors | Description | Case / Controls | Mean | Median | 1^st^ quantile | 3^rd^ quantile | P-value |
| --- | --- | --- | --- | --- | --- | --- | --- |
| No. of cattle | Herd size of cattle at the time of survey | Case (n = 16) | 23.94 | 23 | 15.75 | 26.75 | 0.0006*^1^* |
|  |  | Control (n = 56) | 51.71 | 38 | 24 | 64.4 |  |
| No. of goats | Herd size of goats at the time of survey | Case (n = 8) | 4.25 | 4 | 2 | 5.75 | 0.0648*^1)^* |
|  |  | Control (n = 9) | 21.33 | 8 | 4 | 18.5 |  |
| No. of sheep | Herd size of sheep at the time of survey | Case (n = 14) | 76.86 | 61 | 21.5 | 131.5 | 0.0704*^2)^* |
|  |  | Control (n = 9) | 36.11 | 32 | 21 | 47 |  |
| No. of lamb losses | Number of lamb losses in the last year | Case (n = 8) | 8.5 | 6 | 3.25 | 16.75 | 0.0824*^1)^* |
|  |  | Control (n = 5) | 3.6 | 2 | 2 | 6 |  |

*^1)^ p-value < 0.1, calculated with Mann-Whitney U test*

*^2)^ p-value < 0.1, calculated by t-test*
